# Editorial: The associations of lifestyle factors and behaviors with multimorbidity

**DOI:** 10.3389/fpubh.2023.1227381

**Published:** 2023-06-27

**Authors:** Konstantinos Giannakou, Maria Kyprianidou, Stavri Chrysostomou, Costas A. Christophi

**Affiliations:** ^1^Department of Health Sciences, School of Sciences, European University Cyprus, Nicosia, Cyprus; ^2^Department of Life Sciences, School of Sciences, European University Cyprus, Nicosia, Cyprus; ^3^Cyprus International Institute for Environmental and Public Health, Cyprus University of Technology, Limassol, Cyprus

**Keywords:** multimorbidity, chronic disease, lifestyle behaviors, comorbidity, public health

The global trend of increasing life expectancy and the growing prevalence of multimorbidity, characterized by the coexistence of two or more chronic conditions, present significant challenges for healthcare systems due to their negative impact on health outcomes (1–3). Lifestyle factors, such as nutrition, physical activity, and smoking, have been found to be associated with the development of multiple chronic conditions, indicating their potential role in preventing individual diseases as well as multimorbidity (4, 5). However, the understanding of multimorbidity epidemiology remains limited, primarily due to previous studies focusing on single-disease outcomes and excluding comorbid patients. Therefore, it is crucial to gain a comprehensive understanding of the relationship between various health-related lifestyle factors, behaviors, and multimorbidity within specific populations, to develop effective prevention, diagnosis, and treatment strategies. This Research Topic aims to provide a dedicated platform for researchers to share advancements in investigating the association between health-related lifestyle factors, behaviors, and the development or progression of multimorbidity. The collection includes nine articles that explore different aspects of multimorbidity, such as prevalence, impact on mortality, associated risk factors, and disease combinations across diverse geographical areas and populations.

The study by Arshadipour et al. aimed to determine the prevalence of multimorbidity and common chronic disease combinations, as well as their impact on mortality in men and women aged ≥ 65 years. The authors demonstrated a positive association between multimorbidity and all-cause mortality, and they observed a higher mortality risk in men compared to women. The authors identified hypertension as the most prevalent chronic condition, both in isolation and in combination with other diseases. Notably, the combination of heart disease and diabetes was found to be the most hazardous in both males and females, with a higher risk observed in women compared to men, even when combined with other diseases (Arshadipour et al.).

de Sousa et al. utilized data from the 2019 National Health Survey, a household survey in Brazil, to examine health patterns and their association with chronic kidney disease (CKD) in the Brazilian general population. A total of 90,846 individuals were assessed, and the authors identified using factor analysis three patterns related to health: metabolic factors, behavioral risk factors, and behavioral protective factors. The presence of arterial hypertension, diabetes mellitus, hyperlipidemia, and cardiovascular diseases, collectively referred to as metabolic factors, was found to be associated with the likelihood of presenting CKD. These noteworthy findings underscore the importance of evaluating these metabolic factors together, as individuals often exhibit interconnected factors, highlighting the role of multimorbidity in the development of CKD (de Sousa et al.).

In the longitudinal study conducted by Chen C. et al., the association between multimorbidity and fertility history among middle-aged and older adult women in China was investigated. Data from 10,182 female participants in the China Health and Retirement Longitudinal Study (CHARLS) were utilized. The findings demonstrated that high parity and early childbearing were significantly associated with an increased risk of multimorbidity and a higher number of chronic conditions. Conversely, late childbearing was identified as a significant protective factor against multimorbidity. Moreover, the relationship between fertility history and multimorbidity was influenced by age and the urban-rural dual structure. This study provided valuable data supporting the exploration of sex-specific risk factors for multimorbidity (Chen C. et al.).

Gaskell et al. conducted an online pilot study comparing the lifestyle and health-related characteristics of residents in Qatar and the United Kingdom (UK). The study revealed the co-morbidity of psychiatric disorders in individuals with a BMI > 25 in both Qatar and the UK. Interestingly, the study results indicated no statistically significant associations between comorbidity and several predictors, such as drinking habits, smoking status, physical activity, vegetable consumption etc. Nevertheless, a significant association between sleep perception and comorbidity was found within the UK population (Gaskell et al.).

Hu et al. conducted a study using data from the Diverse Life-Course Cohort study. The goal was to examine the prevalence of common chronic diseases, identify multimorbidity patterns, and explore their diversity across different age groups and cultural backgrounds among adults in the Guangdong province in China. The findings revealed a prevalence of multimorbidity of almost 40%, with the frequency increasing as age advanced. Dyslipidemia was the most common disease, with a prevalence as high as 45%. The study also identified the top three binary multimorbidity combinations being dyslipidemia and hyperuricemia, dyslipidemia and hypertension, and hyperuricemia and hypertension. This exploration of multimorbidity patterns across different cultural backgrounds provides insights into the diversity of health profiles (Hu et al.).

The cross-sectional study conducted by Zheng et al. aimed to investigate the association between multiple lifestyle factors and cardiometabolic multimorbidity. The authors found that participants who engaged in two or more high-risk dietary behaviors, such as overeating and drinking water during meals, had a higher risk of developing cardiometabolic multimorbidity. The authors concluded that identifying individuals with specific high-risk lifestyle behaviors and managing their lifestyle may contribute to improved health outcomes for patients with cardiometabolic multimorbidity (Zheng et al.).

Wang et al. conducted a study using the UK Biobank dataset, to investigate the potential influencing factors associated with the Charlson Comorbidity Index (CCI), a widely used measure of multimorbidity. The study suggested that high waist-to-hip ratio (WHR) and high body mass index were statistically significant predictors of elevated CCI, with WHR having a greater impact. Socioeconomic factors, such as income and the Townsend deprivation index, were also found to be associated with CCI. The authors point out that these factors may interact with each other, emphasizing the need for comprehensive, rational, and robust interventions to promote health (Wang et al.).

The study conducted by Chen M. et al. aimed to investigate the cross-sectional and longitudinal associations between multimorbidity and memory-related diseases (MDs) among middle-aged and older adults in China. The authors utilized data from CHARLS, an ongoing nationally representative study conducted every 2 years, focusing on community-dwelling adults aged 45 years and above. The findings identified significant associations between multimorbidity and MDs, with the strength of the association being more pronounced in middle-aged adults. Specifically, stroke, cancer, heart problems, dyslipidemia, and kidney diseases exhibited stronger cross-sectional relationships with MDs (Chen M. et al.).

Shen et al. utilized data from the Chinese Urban Adults Diet and Health Study to investigate the interaction between overweight/obesity and high glycated hemoglobin (HbA1c) status in relation to elevated high-sensitivity C-reactive protein (hs-CRP) levels among adults in the Chinese population. The study revealed that higher HbA1c levels and overweight/obesity, both independently and in combination, were associated with an increased risk of elevated hs-CRP, particularly among females and younger individuals. The authors concluded that implementing intervention strategies aimed at preventing high blood glucose levels and concurrently managing body weight could play a crucial role in reducing the occurrence of hs-CRP-related diseases (Shen et al.).

Overall, the results of the studies in this Research Topic shed light on the prevalence and impact of multimorbidity in diverse populations. They revealed associations between multimorbidity and increased mortality, identified specific risk factors and disease combinations, and underscored the importance of lifestyle management and early intervention in reducing multimorbidity risk and related diseases. The findings enhance our understanding of multimorbidity and provide valuable insights for the development of targeted interventions and healthcare strategies. We anticipate that this Research Topic will serve as a guide for future researchers to delve further into the intricate nature of multimorbidity, explore novel risk factors, comprehend disease interactions, consider sex-specific and age-specific factors, account for cultural diversity, and conduct more longitudinal and intervention studies. These advancements can contribute to the formulation of effective strategies for preventing, managing, and improving outcomes for individuals affected by multimorbidity.

## Author contributions

KG drafted and wrote the first draft of the manuscript. All authors reviewed, revised, and approved the final manuscript.
